# Imaging the Ancients

**DOI:** 10.1186/1470-7330-15-S1-O21

**Published:** 2015-10-02

**Authors:** Rodney H Reznek

**Affiliations:** 1 Barts Cancer Institute, Barts and The London School of Medicine and Dentistry, Queen Mary University of London, UK

## 

Mummified remains and artefacts, since their discovery, have attracted the interest of scientific investigators. Consequently virtually every paleoimaging modality has been applied to bioarchaeology soon after its technological development. Indeed, the first use of X-rays in mummy investigation was only one year after William Röntgen discovered X-rays in 1895 when, in March 1896, Carl Koenig, a German physicist, published the first X-rays involving mummies, that of an Egyptian mummified cat and the knees of an Egyptian child mummy. The first systematic analysis of a major mummy collection was undertaken at the Field Museum in Chicago in 1931. CT scans of mummies were first carried out in 1977. Not surprisingly, over the years, several problems have been identified in interpreting the images on CT. The presence of many layers of mummy wrapping, organ removal, diagenetic changes, particularly desiccation, may all result in confusion. Special problems in X-ray and CT scanning arise when the diagenetic changes are so massive that the remaining tissues, including bone, are severely degraded - a feature best shown by bog bodies, all of which date to the Northern European Iron Age (approximately 500 BC to 500 AD).

Recent technological advances have made MDCT an especially useful means for *presentation* of findings of anthropological inquiry as exemplified in a brilliant recent exhibition at the British Museum. However, this short lecture will show an almost unique personal experience of imaging of mummies performed over almost a decade in the mid-80s and early 90s. Investigations were undertaken of a bog body discovered in Lindow near Manchester, Muisca bodies discovered in the Andes, and Egyptian mummies from Fayum. All three projects revealed fascinating insights into the lifestyle of these ancients and answered several lines of anthropological inquiry. This presentation will illustrate examples from each of these projects.

## References

[B1] Beckett RG Paleoimaging: a review of applications and challenges Forensic Sci Med Pathol 2014 10 3 423 36 10.1007/s12024-014-9541-z 24682794

[B2] Wade AD Scenes from the past. Multidetector CT of Egyptian mummies of the Redpath Museum Radiographics 2012 32 1235 1250 10.1148/rg.324125704 22787004

[B3] Cox SL A critical look at mummy CT scanning Anat Rec 2015 298 6 1099 1100 10.1002/ar.23149 25998644

[B4] Previgliano CH Radiology evaluation of the Llullaillaco mummies AJR 2003 181 1473 1479 10.2214/ajr.181.6.1811473 14627558

[B5] Reznek RH Stead I.M., Bourke, J.G., Brothwell, D. Computed tomography of Lindow Man Lindow Man, The Body in the Bog 1985 British Museum Publications

